# Delay of transfer from the intensive care unit: a prospective observational analysis on economic effects of delayed in-house transfer

**DOI:** 10.1186/s40001-019-0388-3

**Published:** 2019-09-03

**Authors:** G. Edenharter, D. Gartner, M. Heim, J. Martin, U. Pfeiffer, F. Vogt, K. Braun, D. Pförringer

**Affiliations:** 1 Klinikum rechts der Isar, Technische Universität München, Klinik für Anästhesiologie, Munich, Germany; 2 Klinikum rechts der Isar, Technische Universität München, Klinik und Poliklinik für Unfallchirurgie, Ismaninger Str. 22, 81675 Munich, Germany; 3 School of Mathematics, Cardiff University, Cardiff, UK; 4 Department of Cardiac Surgery, Paracelsus Medical University, Nuremberg, Germany

**Keywords:** Workflow, Delay of ICU discharge, Resource allocation, Bed capacity

## Abstract

**Background:**

Intensive care unit (ICU) capacity is a scant and precious resource in hospitals. Therefore, an optimal occupancy rate as well as detailed occupation planning is of great importance. Most literature deals with admission to the ICU, while only few discuss discharge from the ICU. Specifically, a delay of transfer from the ICU can cause a shortness of beds, jeopardize urgent patient treatment and lead to a decrease in treatment quality as well as economic downsides. This study examined the incidence, costs and reasons for delayed discharge from the ICU and analyzed the influence of the department the patient was admitted to.

**Methods:**

Over the course of 12 months, the discharges of all 1643 patients of two surgical intensive care units of a large academic medical center were analyzed. Delay in minutes and reasons were recorded and translated into financial figures. A univariate logistic regression model was developed to evaluate the impact of length of stay at the ICU, age, gender, subspecialty and specific ICU on the delay of transfer. In a next step, significant factors of the univariate logistic regression were incorporated into a multivariate regression model.

**Results:**

In 326 out of 1312 patients ready for discharge (24.8%), the transfer to the floor was delayed. Time of delay for all patients added up to a total of 265,691 min in 1 year. The application of the internal cost allocation, in which 1 min corresponds to 0.75 Euro cents, led to costs of 199,268 Euros (~ $240,000) for the study period. In 91.7% of the cases, the reason for the delay was the lack of an available or appropriate bed on the regular ward. Multivariate regression analysis revealed that the type of department the patient is admitted to poses a significantly influencing factor for delayed discharge from the ICU.

**Conclusion:**

Delay in discharge from the ICU is a common problem of economic relevance. The main reason is a lack of appropriate floor beds. Patients from certain specific departments are at a higher risk to be discharged with delay. A solution to this problem lies in the focus on the downstream units. A proper use of the scarce resources is to be pursued because of ethical as well as economic reasons in an increasingly aging population.

## Background

Critical care medicine (CCM) is a very important but limited and cost-intensive resource. The cost of CCM cannot be quantified exactly but lies in a range of a triple digit billion $ figure in the United States [1–3]. For this reason, increasing attention has been paid to contain expenditures using these precious resources wisely. There is plenty of literature describing the circumstances resulting in Intensive care unit (ICU)—admission, discharge and triage guidelines [2]. Publications about outflow limitations, however, are rare although those bottlenecks are common in daily practice [4, 5]. Many patients meet the discharge criteria to leave the ICU but transfer is not possible due to different reasons in the downstream units. The aim of this study is to investigate the incidence, costs and reasons for a delayed discharge from the ICU. In a second step, the impact of the subspecialty the patient was admitted to was analyzed.

## Materials and methods

Over the course of 1 year, all patients (*n* = 1643) admitted to two general intensive care units (ICU) of a German 1200 bed academic medical center hospital (Klinikum rechts der Isar, Technical university Munich, Germany) were enrolled in this prospective observational study. Based on consultation of the institutional review board (IRB), informed consent was not necessary because of the purely observational nature of the study. Patient care was not affected by the study and data were analyzed strictly anonymously without individual patient information. The hospital is a level-1 trauma center and the two surgical intensive care units (SICU) consist of 35 beds. Patients are admitted to the ICU mainly from the departments for neurosurgery, abdominal surgery, vascular surgery, trauma surgery and neurology. The wards are run by a 24-h shift staffing by attending intensivists, critical care fellows, residents and highly qualified nurses.

Based on discharge criteria during morning rounds with the attending intensivist, ICU nurse and surgeon in charge, every patient was assessed, and consensus was reached for discharge of the patient to the regular floor. The decision of readiness for discharge had to be taken before 9 a.m. The admission office in charge was informed about the decision and requested to locate an appropriate floor bed. The admission office was constantly aware of the availability of the floor beds. When a bed became available, the patient was transferred to the floor. A patient transfer to the floor was classified as delayed when the patient left the ICU after 2.30 p.m. This time of day was chosen for two reasons:

1. At the Klinikum Rechts der Isar there is a shortage of intensive care beds. For this reason, a triage system for patients who possibly need intensive care treatment was implemented. Those patients are transferred directly after the operation to the post anesthesia care unit and are monitored for their postoperative development. After further assessments and triage, only those patients who are really in need of intensive care treatment are transferred to the ICU. This results in later arrivals at the ICU.
2. The usual practices of the regular wards in our hospital include that patients are often discharged from the regular in the late morning. After discharging the patient from the regular ward activities like cleaning the room on the ward, supplying a clean bed and other preparatory actions start quite late. This results in late supply of regular ward beds.

Based on internal cost allocation, 1 min corresponds to 0.75 Euro cents. This cost calculation is based on a top–down approach for diagnostics, labor, medication and consumables. Overhead costs for administration could not be included in our calculation. Therefore, the top–down approach represents the costs for an average patient and differences between patients cannot be depicted.

Prior to the study, all employees involved in discharging patients identified possible reasons for a delayed discharge. The five reasons identified were:

1. waiting for an appropriate floor bed,
2. waiting for transport service to the floor,
3. delay for any reason caused by the intensive care unit,
4. waiting for an appropriate bed in an external hospital, and
5. waiting for additional diagnostics.

All statistical tests were carried out using the Regression Modeling Strategies (RMS) package in the statistical package R [6] and outliers were removed. In a two-stage approach, first a univariate regression model was developed which controlled for the logarithmic length of stay with basis 2. In a second stage, a multiple Cox model was developed and the significant variable from the univariate analysis were included. Moreover, we controlled for the department to which the patient was admitted. Finally, we controlled for the two different intensive care units.

## Results

During the 12-month duration of the study, 1312 patients out of the 1643 patients met the criteria for discharge and were scheduled for discharge after morning rounds. In 326 out of 1312 patients (24.8%), the transfer to the floor was delayed. Time of delay for all patients added up to a total of 265,691 min which corresponds with an arithmetic mean delay of 815 min per delayed discharge. The application of the internal cost allocation added up to costs of 199,268 Euros (~ $240,000) for the entire study period.

In 91.7% of the cases, the reason for the delay was the lack of an available or appropriate bed on the regular floor. Other reasons are depicted in Table 1.

**Table 1:** Reasons for delay

Reasons for delay	Number	%
Waiting for an appropriate floor bed	299	91.7
Waiting for transport service to the floor	14	4.3
Delay for any reason caused by the intensive care unit	5	1.5
Waiting for an appropriate bed in an external hospital	4	1.2
Waiting for additional diagnostics	4	1.2

In the univariate logistic regression model, length of stay (LOS) on the ICU (*p* < 0.001) and the clinic (Department 1 and Department 2) the patient was admitted to (*p* < 0.001) showed a significant influence on the delay of discharge from the ICU. Age and gender had no significant influence on the delay of discharge. In the multiple logistic regression model with the input of the significant factors—LOS and clinic—the LOS did not have an influence on the delay of discharge (*p* = 0.1051). The only variable remaining with significant influence on the delay in the multivariate regression model was the clinic (Department 1 and Department 2) (*p* < 0.001).

## Discussion

The overall results reveal a serious transfer delay problem with a strong departmental bias within the hospital. The underlying findings confirm the rates of transfer delays in previous studies [4, 5] which also demonstrate that similar problems are evident in countries with different organizational structures. So far, in previous studies, transfer to the ICU and discharge from the ICU have been examined [2, 7]. Criteria of discharge from the ICU, handover itself and its potential improvement have been specified [2, 8], but rates of delay still remain high. In our study, delay was defined as transfer after 2:30 p.m. Though this time of day reflects the usual habits at our hospital, it may seem very late for great majority of the other hospitals where the first patients arrive much earlier. So, compared to the daily practice in other hospitals, there is a considerable underestimation of delay in our study. Under the assumption of a stable rate of delay, a forward displacement of the starting point of delay to 11 a.m. would increase the amount of delay by 68,460 min to a total of 334,151 min. Our economic evaluation is based on the price of 0.75 € for one ICU minute. This corresponds to 199,268 €. In general, it is very difficult to calculate the price for 1 min of intensive care treatment. On the one hand, there are the bottom–up and on the other hand the top–down approaches for the calculation [1]. For the application of the top–down method, data at departmental level are required and therefore are more feasible compared to the bottom–up method which requires data on a patient level. Interpretation of data generated by the top–down approach—which we used—is consequently restricted to the “average patient”. On the other hand, costs for 1 min of intensive care treatment as described in the literature differ massively in different countries. In Europe, prices range from 0.81 € to 1.41 € per minute [9]. Publications in the United States mention even higher costs [10]. Compared to cost for 1 min of intensive care treatment described in literature, the price of 0.75 € is even lower than the bottom of the range for the ICU costs.

In addition, further cost is generally incurred by foregone revenue via canceled operations due to lack of ICU capacity. To specifically calculate the incurred loss is impossible, but a general assumption of one out of an average of four operations per theater not happening on average may yield a 25% reduction in revenue.

The most frequent reason for the delay is a lack of appropriate floor beds in accordance with other studies on this topic [5]. In the above-quoted study of Johnson et al. [5] the ICU–total hospital bed ratio is 15%. In our hospital, this ratio is 5%. At first sight, this appears to render it easier to transfer patients out of the ICU as in relation more floor beds seem available, which, however, has not proven entirely true. So, ICU-to-total hospital beds seem not to be the only relevant predictor for transfer delays. Bagust et al. [11] showed in a stochastic simulation model that in an acute hospital, the risk of no bed being available is observable if occupancy of total hospital beds exceed 85%. During the study period, the average hospital census was 87%. In accordance with the study of Bagust et al., these rates of delay seem not to be surprising. Moreover, Johnson et al. [5] in their study showed evidence of delay in transfer being associated with high hospital census. When planning our study, we did not include the daily census of the examined departments and unfortunately these data are not reliably available on a daily basis in our hospital. Therefore, we cannot make any statements about those correlations. The only data available were the average capacity utilization over the whole year. The Department 1 responsible for the largest fraction of delay and highest percentage of delayed patients displayed a total capacity utilization of 118.5% on their service-specific wards during the observed timeframe. The Department 2 generating significantly less time and cases of delay had a service-specific capacity utilization of 103% in the same time frame. The high utilization of these departments means that a remarkable percentage of their patients are located on non-service-specific floors of different subspecialties. Johnson mentions that a de-specialization of floors could reduce delay of patient transport [5]. This point can neither be supported nor refuted as it was not part of the examination. In the examined hospital, nearly 100% of the patients are transferred to the service-specific floor. According to the departments’ policy, a subsequent transfer to non-service-specific floors is only performed if patients are ready for a timely hospital discharge. This allows the conclusion that capacity utilization of the service-specific floor seems to be the influencing factor for delay. De-specialization of floors may indirectly influence the delay by creating space on service-specific floors. Specifically, less care-intensive patients could be transferred to de-specialized floors, allowing for more space on specialized wards. This effect can be observed in large university hospitals often characterized by a generally high rate of specialization and accompanying specific infrastructure. One logical solution would be the creation of more beds on service-specific floors to reduce their average occupancy rates. As this performance figure is a major point in discussions with the management board, it is to be questioned whether the creation of new beds and therefore lower occupancy rates would be accepted as a solution to the problem. In general, beds tend to be shifted between departments but only rarely can be created de novo. This point cannot be influenced by the intensivist.

The univariate regression model revealed that age and gender did not have a significant impact on the delay. On the other hand, the department, the specific intensive care unit and a prolonged stay on the ICU put the patients at risk of being transferred with a delay. In the multiple regression model with influence variables department, type of intensive care unit and a prolonged stay on the ICU only the department remained a significant factor. The correlation between high capacity utilization and a delayed discharge is described above. In summary, the effective solutions for the described problems are found within the departments. Young et al. [12] present an interventional study in which the ICU throughput was significantly improved. A multidisciplinary workgroup with representatives from the ICU, the OR, PACU as well as surgical coordinators, an administrative fellow and a lean six sigma consultant was implemented. Their main interventions among others were the early identification of clinically stable patients, a goal of transferring two patients to the floor before 10 a.m. and collaboration with downstream wards to achieve the same targets. The last point can be regarded as the most important as every discharge from the ICU is dependent on available space on the regular floor. This means that in these cases, whenever there is the outlook of a transfer to the floor, identifiable communication must start to discuss reasons and causes which prevent a timely and safe discharge from the ICU.

## Limitation

A limitation of our study was that the expression “no bed available” was not objectively evaluated in regard to other reasons like shortness of nurses or others to prevent a safe monitoring of the patient on the regular floor. Another limitation was that we did not link the daily hospital census of the patients of a distinct clinic to the delay. Therefore, we cannot claim if the daily census has any influence on the incidence of a delay.

## Data Availability

Further detailed data are available upon request from the first author, Dr. med. Günther Edenharter.
